# Identifying stigmatizing language in clinical documentation: A scoping review of emerging literature

**DOI:** 10.1371/journal.pone.0303653

**Published:** 2024-06-28

**Authors:** Veronica Barcelona, Danielle Scharp, Betina R. Idnay, Hans Moen, Kenrick Cato, Maxim Topaz

**Affiliations:** 1 Columbia University School of Nursing, New York, New York, United States of America; 2 Department of Biomedical Informatics, Columbia University, New York, New York, United States of America; 3 Department of Computer Science, Aalto University, Aalto, Finland; 4 University of Pennsylvania School of Nursing, Philadelphia, Pennsylvania, United States of America; State University of New York at Oswego, UNITED STATES

## Abstract

**Background:**

Racism and implicit bias underlie disparities in health care access, treatment, and outcomes. An emerging area of study in examining health disparities is the use of stigmatizing language in the electronic health record (EHR).

**Objectives:**

We sought to summarize the existing literature related to stigmatizing language documented in the EHR. To this end, we conducted a scoping review to identify, describe, and evaluate the current body of literature related to stigmatizing language and clinician notes.

**Methods:**

We searched PubMed, Cumulative Index of Nursing and Allied Health Literature (CINAHL), and Embase databases in May 2022, and also conducted a hand search of IEEE to identify studies investigating stigmatizing language in clinical documentation. We included all studies published through April 2022. The results for each search were uploaded into EndNote X9 software, de-duplicated using the Bramer method, and then exported to Covidence software for title and abstract screening.

**Results:**

Studies (N = 9) used cross-sectional (n = 3), qualitative (n = 3), mixed methods (n = 2), and retrospective cohort (n = 1) designs. Stigmatizing language was defined via content analysis of clinical documentation (n = 4), literature review (n = 2), interviews with clinicians (n = 3) and patients (n = 1), expert panel consultation, and task force guidelines (n = 1). Natural language processing was used in four studies to identify and extract stigmatizing words from clinical notes. All of the studies reviewed concluded that negative clinician attitudes and the use of stigmatizing language in documentation could negatively impact patient perception of care or health outcomes.

**Discussion:**

The current literature indicates that NLP is an emerging approach to identifying stigmatizing language documented in the EHR. NLP-based solutions can be developed and integrated into routine documentation systems to screen for stigmatizing language and alert clinicians or their supervisors. Potential interventions resulting from this research could generate awareness about how implicit biases affect communication patterns and work to achieve equitable health care for diverse populations.

## Introduction

Racial and ethnic disparities in health care access, treatment, and outcomes have been documented for decades [1]. Prior studies have shown that concerns expressed by Black patients are more likely to be dismissed or ignored than White patients [2]. This differential treatment has been observed among Black and African American patients leading to disparities in outcomes, [1, 3, 4] and specifically in the treatment of cardiovascular diseases, [5] pain, [6] and breast cancer [7]. Racism occurring on the structural, interpersonal, or cultural levels has been identified as the primary reason for disparities in health outcomes [8]. Researchers have examined clinician biases by studying racial bias in patient-clinician interactions, finding that stereotyping and lack of empathy towards patients by race influenced health care outcomes [9].

Stigmatizing language has been defined as language that communicates unintended meanings that can perpetuate socially constructed power dynamics and result in bias [10]. Recent studies suggest that racial biases may also be identified by examining stigmatizing language in clinician notes documented in the electronic health record (EHR) [11–14]. Racial differences in documentation patterns may reflect unconscious biases and stereotypes that could negatively affect the quality of care [14]. Examples of stigmatizing language may include the use of quotations to identify disbelief in what the patient is reporting, questioning patient credibility, sentence construction that implies hearsay, and the use of judgment words [13]. Stigmatizing language in clinical notes has been associated with more negative attitudes towards the patient and less effective management of patient pain by physicians [14].

### Objective

It is unknown to what extent and how stigmatizing language has been studied in healthcare settings, and study designs and foci differ. Emerging studies have used traditional qualitative methods, including interviews with patients and clinicians. Other research has used natural language processing (NLP), a computer science-based technique that helps extract meaning from large bodies of text, to quantify how EHR notes reflect stigmatizing language by race and ethnicity. The purpose of this scoping review was to identify, describe, and evaluate the presence and type of stigmatizing language in clinician documentation in the literature.

### Design

A scoping review was chosen instead of a systematic review as the purpose was to identify and map the emerging evidence [15]. This review was conducted using PRISMA-ScR guidelines for scoping reviews [16].

## Materials & methods

### Search strategy

The authors discussed the selection and coverage of three concepts (i.e., stigmatizing language, clinician, and clinical documentation) for review based on the research question. For purposes of the current study, the concept of “clinician” includes physicians and nurses. We searched PubMed, Cumulative Index of Nursing and Allied Health Literature (CINAHL), and Embase databases in May 2022 to identify studies investigating stigmatizing language in clinical documentation. We also conducted an updated hand-search of the IEEE Explore database for articles published through April 2022. However, we did not identify additional articles that met inclusion criteria and were not already included in our review. The results for each search were uploaded into EndNote X9 software, de-duplicated using the Bramer method [17], and then exported to Covidence software for title and abstract screening. The search strategy is detailed in S1 Table.

### Inclusion criteria

**Fig 1:**
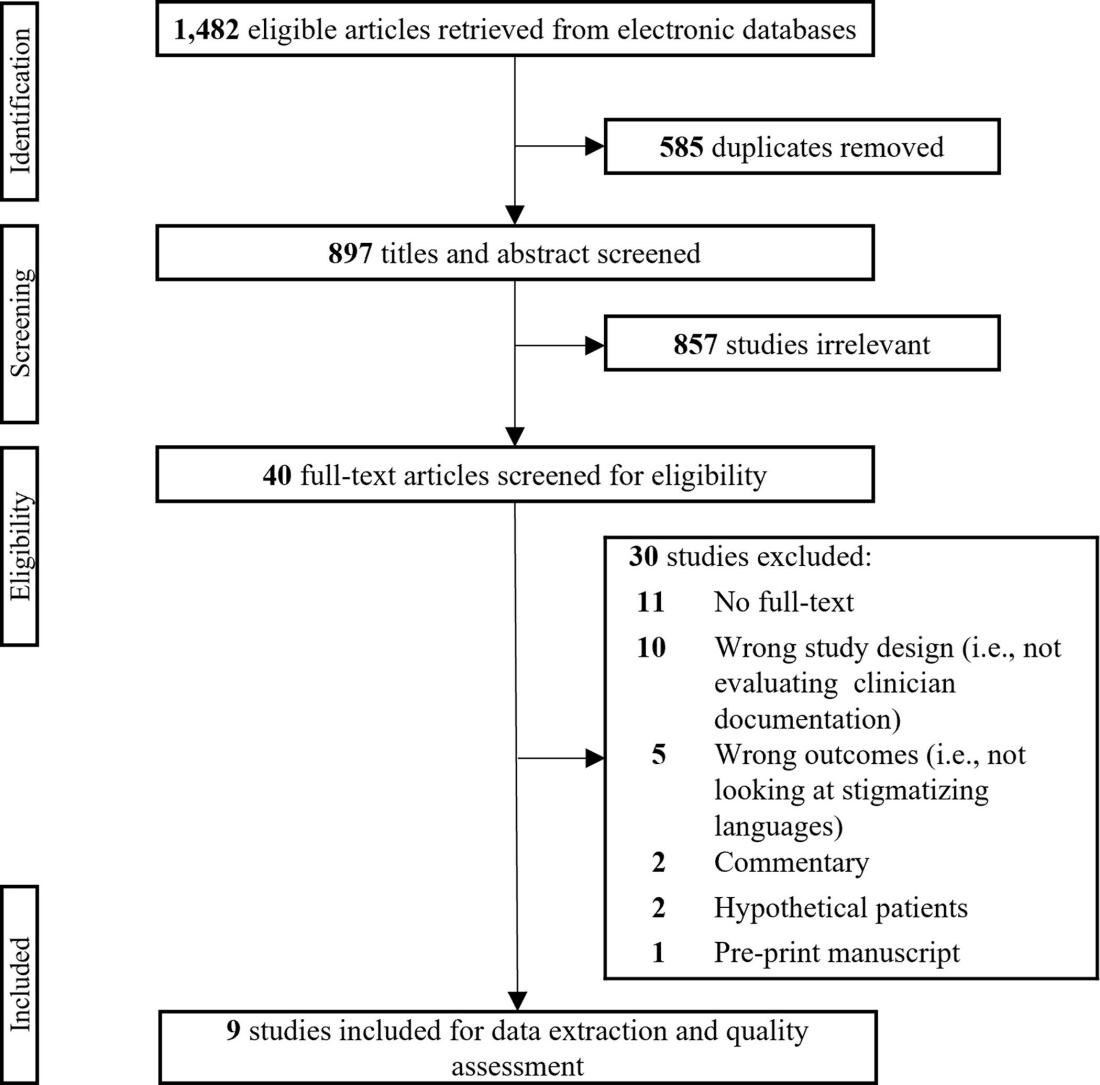
PRISMA-Scr diagram.

The initial search yielded 1,482 articles for review. After de-duplication, 897 articles were included for title and abstract screening. Two authors (BI, DS) independently screened all articles by title and abstract and documented reasons for exclusion, when applicable. Studies were included if they investigated stigmatizing language in clinical documentation. Studies that looked into stigmatizing language with patient-provider interaction that did not include documentation (e.g., verbal communication) were excluded. Articles not in English, review articles, editorials, commentaries, and articles without full-text availability were also excluded. The same reviewers independently assessed all potentially relevant articles in the full-text review to comprehensively determine eligibility for inclusion, as well as searching reference lists for additional articles. Discrepancies were discussed with the team to achieve consensus. From the 40 articles included for full-text review, nine articles were included for final synthesis (Fig 1).

### Data extraction and quality assessment

Relevant information categories from each included article were extracted by two authors (BI, DS). Two other co-authors with expertise in health informatics (MT, HM) reviewed and validated all the extracted data elements. These information categories included: authors, year of publication, study aim and design, clinical setting, data source, clinician specialty, clinical note type (when available), study population, number of clinical notes used, data analysis approach, outcomes, and stigmatizing language identified. The Mixed Methods Appraisal Tool (MMAT) [18] was used to evaluate study quality and the risk of bias in the included articles.

## Results

Nine articles meeting all inclusion criteria were included in this scoping review (Table 1). Overall, study designs (N = 9) included cross-sectional (n = 3), [11–13] qualitative (n = 3), [19–21] mixed methods (n = 2), [22, 23] and retrospective cohort (n = 1) [24]. Studies took place in exclusively inpatient (n = 3) [12, 19, 24] or outpatient (n = 4) [13, 21–23] settings. One study was conducted in an emergency department (ED) (n = 1), [20] and another included participants from inpatient, outpatient, and ED settings (n = 1) [11]. In terms of patient population, six focused on general medicine, [11–13, 19, 21, 23] and one article each on oncology, [22] psychiatry, [24] and pediatrics [20].

**Table 1:** Characteristics of articles examining stigmatizing language in clinician notes.

Study	Purpose	Study Design	Clinical Setting and country	Type and Number of Notes	Type and Number of Clinicians	Type of stigmatizing language	Total Number of Unique Patients, % Race/Ethnicity	Methods	Results
Alpert et al., 2019	To establish a baseline of the linguistic characteristics and patterns used in notes.	Mixed methods	Outpatient, oncology, Virginia, USA	Assessment, plan, interval history, impression, and free summary; 207	Oncologists; 13	Stigmatized and sensitive words: “patient”, distress/ stress, mental, alcohol, depression	Not specified	Qualitative in-depth respondent interviews (grounded theory) and descriptive statistics of clinician notes. NLP method: Linguistic Inquiry and Word Count (LIWC) to identify language patterns.	Notes frequently contained a negative emotional tone, low authenticity, high cloud, and high analytical writing styles. The identified themes related to stigmatizing language were audience uncertainty, censorship due to social terms, and the potential to help or hurt relationship building.
Beach et al., 2021	To identify language that conveys disbelief and discern racial and gender differences in the use of this language.	Cross sectional	Outpatient, internal medicine, academic medical center, USA	Physician notes; 9521	Physicians; 165	Three linguistic features suggesting disbelief were used: quotes, judgment words, and evidentials (patients’ symptoms or experience reported as hearsay)	N = 3374; 26% White/Caucasian, 74% Black/African American	Content analysis of 600 randomly selected notes to identify linguistic features. NLP system: Custom Aho-Corasick algorithm with a Trie-based data structured implemented in FlashText. NLP method: Regular expression (removing extra space) and spaCy (sentence and word tokenization, part of speech tagging and dependency parsing)	Stigmatizing language was defined as judgment words conveying negative judgement, quotes suggesting disbelief, and evidentials discrediting patients’ reports. EHR notes written about Black patients compared to White patients had higher odds of including at least one judgment word, at least one quote, and more evidentials.
Fernandez et al., 2021	To understand how many patients feel judged or offended due to something they read in outpatient notes and why.	Mixed methods	Outpatient, hospital offices and community practices in 3 health systems, Massachusetts, Washington, Pennsylvania, USA	Not applicable	Not specified	Judgmental or offensive language: disrespect, errors and surprises, labeling, and other themes	N = 22959; 79% White, 2% Black, 5% Asian. 6% Other/ multiracial; 6% missing; 3% Hispanic/Latino ethnicity.	Quantitative analysis of 2 dichotomous questions (feeling judged and offended yes/no), and qualitative thematic analysis of free-text responses on what patients found judgmental or offensive.	Patients reported feeling: judged [608(25.2%)], offended [748 (31.0%)], and both judged and offended [1,055(43.8%)]. Themes identified related to stigmatizing language: labeling and disrespect
Himmelstein et al., 2022	To identify the frequency of stigmatizing language patterns by diagnosis (diabetes, substance use disorder, chronic pain) race, ethnicity, and clinician characteristics.	Cross sectional	Inpatient, Massachusetts, USA	Hospital admission notes; 48651	Physicians, physician assistants, nurse practitioners, nurse midwives, nurse anesthetists; N = 1932	Labeling, blaming, or invoking danger or peril in patients with diabetes, substance use disorder, and chronic pain	N = 29783; 2.8% Hispanic; 3.5% non-Hispanic Asian; 8.4% non-Hispanic Black; 63.6% non-Hispanic White, 4.7% Other	NLP method: tokenized free text of each note into unigrams and bigrams Standardized terminology: ICD-10 codes; used guidelines from task forces from the Association of Diabetes Care and Education Specialists, the American Diabetes Association, National Institute on Drug Abuse (NIDA), studies related to pain to identify stigmatizing language	Diagnosis-specific stigmatizing language was found in 599 notes (6.9%) for patients with diabetes, 209 (3.4%) for patients with substance use disorders, and 37 (0.7%) for patients with chronic pain. Notes for non-Hispanic Black patients had a greater probability of including stigmatizing language than non-Hispanic Whites.
Hoover et al., 2021	To explore how addiction consultation services affect patients’ and providers’ experiences with stigma in the hospital setting.	Qualita-tive	Inpatient treatment of patients with substance use disorder, Colorado, USA	Not applicable	Hospitalists, nurses, social workers, pharmacists; N = 62	Transmission of stigma in inter-personal and patient-provider interactions	N = 20; 75% White, 25% Hispanic	Thematic analysis of 6 focus groups and 8 interviews with clinicians and 20 interviews with patients	Themes identified related to stigmatizing language: clinician documentation unintentionally or intentionally perpetuates stigma among clinicians and leads to anticipated stigma among patients
Landau et al., 2022	To explore considerations for generating am EHR-based phenotype of child abuse and neglect in EDs including implications for racial bias reduction.	Qualita-tive	Pediatric ED, Northeast USA	Not specified	Social workers, pediatricians, respiratory therapists, nurses, physician assistants; N = 20	Racial bias in documen-tation of child abuse and neglect, including non-compliance, difficult patient, pain-seeking behavior	Not specified	Semi-structured interviews	Themes identified related to stigmatizing language: challenges in diagnosing child abuse/neglect, differences in documentation styles by discipline, and use of documentation to identify potential racial bias
Martin et al., 2020	To examine the language used by mental health forensic nurses and describe the characteristics of these words (neutral, ambiguous, negative).	Retro-spective cohort chart review	Inpatient psychiatric unit, Ontario, Canada	Nursing progress notes; 1608	Nurses; N = 55	Positive words, template words, and ambiguous or misleading words	Not specified	Human chart review to identify adjectives used to describe patients, and verbs representing staff and patient actions. Valence scores were assigned to each identified word (1 = negative, 5 = neutral, 9 = positive) Quantitative analyses to compare independent groups.	242 unique words were identified. 8 words had a negative valence score: compliant, seclusive, irritable, agitated, restless, angry, dismissive, anxious. Ten sets of nursing notes had a negative valence score, 45 sets of nursing notes had a positive valence score. No difference in valence score related to age range, experience, profession, gender.
Park et al., 2021	To identify language in health records that may show negative and positive attitudes toward the patient.	Qualita-tive	Outpatient, internal medicine, urban medical center, USA	Physician notes; 600	Attendings and residents; N = 138	Patterns of positive and negative language in EHR docu-mentation	N = 507; 80% Black/African American, 15% White	Qualitative content analysis (inductive approach) of unstructured free text data in EHRs; extracted words/phrases that had positive or negative valence	Identified 5 categories of negative language: questioning patient credibility, disapproval, stereotyping, difficult patient, unilateral decisions Identified 6 categories of positive language: compliment, approval, self-disclosure, minimizing blame, personalize, bilateral decision making
Sun et al., 2022	To determine whether providers’ use of negative patient descriptors differed by patient race or ethnicity	Cross sectional	ED, inpatient, outpatient, urban academic medical center, Illinois, USA	History and physical notes; 40113	“Medical providers”; N = 33142 encounters, not specified how many per clinician	Expert panel and literature review selected fifteen descriptors for analysis: (non-)adherent, aggressive, agitated, angry, challenging, combative, (non-)compliant, confront, (non-)cooperative, defensive, exaggerate, hysterical, (un-)pleasant, refuse, and resist.	N = 18459; 29.7% White, 60.6% Black, 6.2% Hispanic or Latino, 3.5% Other	Literature search and expert panel consult to determine 15 negative descriptors: (non)adherent, aggressive, agitated, angry, challenging, combative, (non)compliant, con-front, (non)cooperative, defensive, exaggerate, hysterical, (un)pleasant, refuse, and resist. Used NLP to standardize text data and split notes into sentences. Categorized the use of each descriptor (negative, positive, out of context) Machine learning: divided labeled sentences into 66% training set, 33% testing set; trained model interpreted the sentences and predicted their context as negative, positive, or out of context. Multilevel mixed effects logistic regression models to determine the odds of a negative patient descriptor in each note as a function of race or ethnicity	Model accurately predicted the context of a sentence with a macro average value F1 of 0.935. Most commonly used descriptors were "refused" (n = 1461), "(not) adherent" (n = 605), "(not) compliant”(n = 561), and “agitated”(n = 409). Black patients had 2.54 times the adjusted odds of having one or more negative descriptors in the EHR. Black race was associated with 5.6 additional negative notes per 100 patients relative to White race.

Methods for measuring and defining stigmatizing language varied by study. Specifically, stigmatizing language was identified via interviews with clinicians [19, 20, 22] and patients, [19] content analysis of clinical documentation, [13, 21, 23, 24] literature review, [11, 12] expert panel consultation, [11] and task force guidelines from relevant professional organizations [12]. Definitions of stigmatizing language or bias varied as well by study, with most studies focusing on discipline-specific words communicating judgment or negative bias (Table 1). Stigmatizing language often included stereotyping by race and ethnicity. An example found in clinician documentation in the EHR was in the form of quotes highlighting “unsophisticated” patient language, i.e., “…patient states that the wound ‘busted open’” [21]. Another study found that physician notes written about Black patients had up to 50% higher odds of containing evidentials (language used by the writer questioning the veracity of the patient’s words) and stigmatizing language than those of White patients [13]. Similarly, physicians documented more negative feelings such as disapproval, discrediting, and stereotyping toward Black patients than White patients [21].

Often, clinical documentation studied was in the form of clinical notes. The most commonly analyzed clinical notes included those documented by physicians (n = 3), [12, 13, 22] followed by nurses (n = 1), [24] advanced practice providers (n = 1), [12] and interdisciplinary team members including radiologists, respiratory therapists, nutritionists, social workers, case managers, and pharmacists (n = 1). Sun et al. examined history and physical notes written by medical providers, although no further detail about the type of providers was specified [11].

Reporting of race and ethnicity of study participants varied widely. In three studies, race was not specified at all, [20, 22, 24] or studies reported only White and Black participant races (n = 2) [13, 21]. Two studies described findings by race and ethnicity, including Black (or African American), Hispanic, White, and Asian categories [12, 23]. The remaining studies either reported race and ethnicity as: White, Black or Hispanic, [11] or White or Hispanic [19].

Studies that conducted interviews focused on how clinical notes were written and may be interpreted by patients, [22] barriers and facilitators to providing care, [19] patients’ perceptions of their hospitalization, [19] and clinician insights on racial bias and EHR documentation [20]. Qualitative themes identified related to stigmatizing language included a reluctance to describe patients as “difficult” or “obese” due to the social stigma attached to common medical language, [22] intentional and unintentional perpetration of stigma in clinical notes, [19] and identification of potential racial bias through documentation [20].

In terms of methods, four studies used NLP [11–13, 22] to extract terms from clinical notes matching those in predefined vocabularies of stigmatizing language terms. After NLP, statistical analyses were conducted to calculate and compare the odds of stigmatizing language occurrence among different patient populations. Two of the NLP-based studies used Linguistic Inquiry and Word Count (LIWC: a standardized vocabulary of terms), while others created their own hand-crafted vocabularies. One of the studies that involved the use of NLP [11] developed a machine learning classifier that would automatically detect stigmatizing language. This was the only study that measured the accuracy of automated NLP-based stigmatizing language detection and found it very accurate (F1 score = 0.94).

Despite a wide variety of clinical settings in the reviewed studies, negative language, bias, racial bias, or stigmatizing language was identified in clinician attitudes and/or documentation across all studies that could negatively impact patient perception or outcomes. Disparities in stigmatizing language use in the EHR were evident by race and ethnicity both in clinician interviews [20, 22, 24] and analyses of clinical notes [11–13, 19, 21, 23]. There may be discipline-specific stigmatizing language and terms [i.e., addiction [19]] and paternalistic attitudes that state that clinical notes are for clinician communication and not for patients to read [i.e., oncology [22]] that warrant further investigation.

In Table 2, results of the study quality assessments are presented. All studies asked clear research questions and collected data to address the research questions. Among quantitative studies (n = 4), three met all five criteria for quality, and the remaining study did not adequately describe measurement, confounders, or intervention fidelity. The qualitative studies (n = 3) met the criteria for four of five quality components assessed, with two studies lacking an explicit discussion of the qualitative approach. Neither mixed methods studies (n = 2) met all quality criteria, as one did not include an adequate rationale for using this design, the other study did not discuss inconsistencies between quantitative and qualitative results, and both did not adhere to all criteria for quantitative and/or qualitative methods.

**Table 2:** Quality assessment for studies examining stigmatizing language in clinician notes.

	Quantitative Studies (n = 4)				Qualitative Studies (n = 3)			Mixed Methods Studies (n = 2)	
**MMAT Questions**	Beach et al., 2021	Himmelstein et al., 2022	Martin & Stanford, 2020	Sun et al., 2022	Hoover et al., 2021	Landau et al., 2022	Park et al., 2021	Alpert et al., 2019	Fernandez et al., 2021
**All Studies**									
Are there clear research questions?	Yes	Yes	Yes	Yes	Yes	Yes	Yes	Yes	Yes
Do the collected data allow to address the research questions?	Yes	Yes	Yes	Yes	Yes	Yes	Yes	Yes	Yes
**Quantitative Studies**									
Are the participants representative of the target population?	Yes	Yes	Yes	Yes	n/a	n/a	n/a	No No table or explanation of detailed demographics of the clinicians	Yes
Are the measurements appropriate regarding both the outcome and intervention (or exposure?)	Yes	Yes	No Unclear how words were extracted; no mention of interrater agreement	Yes	n/a	n/a	n/a	Yes	No The questions were not validated; many patients did not distinguish between “offended” and “judged”
Are there complete outcome data?	Yes	Yes	Yes	Yes	n/a	n/a	n/a	Yes	No 21% response rate
Are the confounders accounted for in the design and analysis?	Yes	Yes	No Did not examine patient characteristics	Yes	n/a	n/a	n/a	No No mention of confounders; limited confidence that LIWC understands context, especially with the word “patient”	No Only frequency analyses (Chi-Square) conducted
During the study period, is the intervention administered (or exposure occurred) as intended?	Yes	Yes	No Participants were informed that notes would be analyzed so participants may have changed the way in which they document	Yes	n/a	n/a	n/a	Yes	Yes
**Qualitative Studies**									
Is the qualitative approach appropriate to answer the research question?	n/a	n/a	n/a	n/a	No No explicit discussion of qualitative approach	No No explicit discussion of qualitative approach	Yes	Yes	Yes
Are the qualitative data collection methods adequate to address the research question?	n/a	n/a	n/a	n/a	Yes	Yes	Yes	Yes	No No report of data saturation
Are the findings adequately derived from the data?	n/a	n/a	n/a	n/a	Yes	Yes	Yes	Yes	Yes
Is the interpretation of results sufficiently substantiated by data?	n/a	n/a	n/a	n/a	Yes	Yes	Yes	Yes	Yes
Is there coherence between qualitative data sources, collection, analysis, and interpretation?	n/a	n/a	n/a	n/a	Yes	Yes	Yes	Yes	Yes
**Mixed Methods Studies**									
Is there an adequate rationale for using a mixed methods design to address the research question?	n/a	n/a	n/a	n/a	n/a	n/a	n/a	Yes	No Vague statement that they build on quantitative findings with qualitative analysis but no further details provided about rationale
Are the different components of the study effectively integrated to answer the research question?	n/a	n/a	n/a	n/a	n/a	n/a	n/a	Yes	Yes
Are the outputs of the integration of qualitative and quantitative components adequately interpreted?	n/a	n/a	n/a	n/a	n/a	n/a	n/a	Yes	Yes
Are divergences and inconsistencies between quantitative and qualitative results adequately addressed?	n/a	n/a	n/a	n/a	n/a	n/a	n/a	Yes	No Quantitative analysis examined if patients felt judged or offended (yes/no)Qualitative analysis only investigated free text for those who reported feeling judged/offended
Do the different components of the study adhere to the quality criteria of each tradition of the methods involved?	n/a	n/a	n/a	n/a	n/a	n/a	n/a	No Does not adhere to all criteria for quantitative methods	No Does not adhere to all criteria for quantitative and qualitative methods

## Discussion

In this review, we identified the types and frequency of stigmatizing language in EHR notes, establishing an underpinning for future research on the correlation between communication patterns and outcomes (i.e., hospitalization, mortality, complications, disease stability, symptom control). With continuous advancements in the field of NLP, we believe that these methods (including deep learning-based methods) will be essential tools in future stigmatizing language studies.

It is crucial to evaluate NLP-based system performance to ensure accurate concept identification and reliable results; however, this was only done in one study [11]. Further studies that use NLP are needed that evaluate the accuracy of the resulting NLP systems and to ensure stigmatizing language is identified correctly. The two studies reviewed here that used NLP did not assess clinical relevance, limiting their findings. In addition to accurate stigmatizing language identification, clinical relevance must be assessed to determine to what extent NLP systems are useful for predicting the association between language use and clinical outcomes. Finally, there is a gap in the literature for NLP-specific bias assessment. There is a need for further development of NLP for identifying stigmatizing language, as these methods may not detect all stigmatizing language, and outcomes may be driven by the level of bias among annotators. Quality from training data is vital in algorithm development, and more research should be done describing biases of people performing annotation. This type of acknowledgment is increasingly common in journals where authors are required to submit positionality statements, however, we suggest that this go further for annotators, as life experiences influence assessments of whether bias or stigma is present. We did not do a specific evaluation of the NLP-only studies, due to the small number. However, further studies should be done to evaluate the quality of NLP studies and the validity of NLP results. Specific criteria for this domain should be developed.

The identification of stigmatizing language use in EHR notes is vital as this language may foster the transmission of bias between clinicians and may represent a value judgment of the intrinsic worth assigned to a patient [11]. Further, with the passage of The 21^st^ Century Cures Act in the US, federal policy now requires the availability of clinical notes to patients [25]. Clinical notes that reflect clinician bias may harm the patient-clinician relationship and hinder or damage the establishment of trust required for positive interactions in health care settings. Medical mistrust is a persistent problem contributing to delays in seeking care and widening disparities in disease outcomes for many vulnerable populations, [26] hence efforts are needed to improve the current situation.

Definitions of stigmatizing language varied in the studies reviewed, and also represent an area for future research. Stigmatizing language may best be defined by the vulnerable populations at risk, in partnership with researchers. Further, discipline-specific language should be discussed and agreed upon, as this may vary by patient population. For example, guidelines have been suggested for addressing the intersectional nature of language in the care of birthing people [27].

Three studies reviewed here did not specify race or ethnicity of their clinician and patient participants [20, 22, 24]. This is a significant issue as patient-clinician race discordance has been associated with increased risk of mortality [28]. Racial concordance, however, does not necessarily lead to better communication as perceived by patients [29]. Given the inconsistency in reporting of race and ethnicity in the reviewed studies, future research in this area should carefully operationalize and define race and ethnicity variables extracted from the EHR. In addition, studies whose primary focus was to identify bias did not blind for patient race, as in many cases race was considered a primary predictor or variable of interest. This underscores an important gap in the literature for NLP-specific bias assessment. Blinding sensitive categories when screening records for bias may improve validity of outcome ascertainment, however, it is often necessary for reviewers to rely on context and include categories such as race and ethnicity when evaluating for stigmatizing language.

The measurement of race is a contentious issue in many medical and scientific disciplines, and though it is a social construction with no biological basis, it remains an indicator of likelihood of encountering racism and racist structures that lead to health disparities. EHR demographic data have been shown to have several quality issues, with some studies indicating that data from Latinos having higher rates of misclassification than other racial/ethnic groups [30]. It is important to consider who enters race and ethnicity data in the EHR, as patient self-identification is often used as the “gold-standard” in research, yet the patient’s apparent phenotype may be an even more important predictor of clinician perception and subsequent clinical documentation. Indeed, recent work has identified that patient race can be predicted using machine learning algorithms applied to other clinical indicators from the EHR [31–33]. From a validity and reliability perspective, researchers must align their methodological definition of race and ethnicity with the stated research objectives. Further, consistent definitions of racial and ethnic categories are essential to identifying associations between stigmatizing language use and patient outcomes as future studies developing interventions are considered. Future research should include larger proportions of minoritized patient and clinician participants to elucidate these issues further, and examine the underlying factors associated with poorer outcomes in various healthcare settings.

Finally, six of the studies reviewed [12, 13, 19–22] included physicians, and many included other health care provider types (i.e. nurses, respiratory therapists, pharmacists, etc.) either alone [24] or in addition to physician notes/participants [12, 19, 20]. Limited information was provided about the type of notes that were analyzed. Further detail about the type of clinicians and notes would allow for the identification of what other disciplines are reading or writing to draw conclusions about the transmission of bias over the trajectory of patient care.

There are several opportunities for policy change to address the use of stigmatizing language in clinical documentation. First, stigmatizing language can be identified automatically with NLP. NLP-based solutions can be developed and integrated into routine documentation systems to screen for stigmatizing language and alert clinicians or their supervisors. Previously published instances of flags in EHR documentation have provided evidence of improved outcomes of care, including in diagnosis of stroke, increasing health care access for patients at risk of suicide, and improving community rates of Hepatitis C screening for those at high risk [34–36]. To our knowledge, NLP findings of stigmatizing language use in the EHR has not yet been applied to clinical practice, identifying a need for future research that could lead to practice and policy change.

Second, clinicians’ less than optimal working conditions may contribute to burnout and negative language use toward patients. One study found that resident physicians who reported higher levels of burnout had greater explicit and implicit racial biases [37]. Individually-focused interventions for clinicians, such as mindfulness training, have also been suggested as a method to reduce bias in clinical care, [38] but have yet to be evaluated. A study carried out on nurses in Taiwan suggested that workplace burnout was associated with poorer patient care outcomes, though stigmatizing language was not examined [39]. The COVID-19 pandemic has also contributed to moral injury for nurses, affecting patient care [40]. Burnout does not foster an environment where clinicians can foster and sustain empathy for patients, and empathy is a critical component of reducing bias and building support for antiracism efforts to reduce inequities [41, 42] Antiracism and bias efforts in hospitals should include analyzing if clinician burnout is associated with stigmatizing language use in EHR documentation, and if it reinforces bias between clinicians, potentially contributing to health inequities.

In summary, this review highlights a new and promising application of qualitative research and NLP to clinical documentation in the study of racial and ethnic disparities in health care. We suggest that further research be done applying NLP to identify stigmatizing language, with the ultimate goal of reducing clinicians’ stigmatizing language use in health documentation. By improving identification of stigmatizing language through NLP and other methods, potential interventions can be developed to generate awareness and design educational interventions about how implicit biases affect communication patterns and work to achieve equitable health care for diverse populations.

## Supporting information

S1 Checklist Preferred Reporting Items for Systematic reviews and Meta-Analyses extension for Scoping Reviews (PRISMA-ScR) checklist. (DOCX)

S1 File Data availability statement. (DOCX)

S1 Table Search strategy. (DOCX)
