# Supplementary material for: Identifying stigmatizing language in clinical documentation: A scoping review of emerging literature
Source: PLoS One. 2024 Jun 28;19(6):e0303653. doi: 10.1371/journal.pone.0303653 (PMC11213326; doi:10.1371/journal.pone.0303653)
Supplement: S1 File — (DOCX) [file pone.0303653.s002.docx]

**Data availability statement**

The articles included in this review are cited below with the doi where each article can be found.

Alpert JM, Morris BB, Thomson MD, Matin K, Geyer CE, Brown RF. OpenNotes in oncology: oncologists' perceptions and a baseline of the content and style of their clinician notes. Translational behavioral medicine. 2019;9(2):347-56. doi: 10.1093/tbm/iby029.

Beach MC, Saha S, Park J, Taylor J, Drew P, Plank E, et al. Testimonial Injustice: Linguistic Bias in the Medical Records of Black Patients and Women. Journal of general internal medicine. 2021;36(6):1708-14. doi: 10.1007/s11606-021-06682-z.

Fernández L, Fossa A, Dong Z, Delbanco T, Elmore J, Fitzgerald P, et al. Words Matter: What Do Patients Find Judgmental or Offensive in Outpatient Notes? J Gen Intern Med. 2021;36(9):2571-8. doi: 10.1007/s11606-020-06432-7.

Himmelstein G, Bates D, Zhou L. Examination of Stigmatizing Language in the Electronic Health Record. JAMA Netw Open. 2022;5(1):e2144967. doi: 10.1001/jamanetworkopen.2021.44967.

Hoover K, Lockhart S, Callister C, Holtrop JS, Calcaterra SL. Experiences of stigma in hospitals with addiction consultation services: A qualitative analysis of patients' and hospital-based providers' perspectives. Journal of substance abuse treatment. 2022;138:108708. doi: 10.1016/j.jsat.2021.108708.

Landau AY, Blanchard A, Cato K, Atkins N, Salazar S, Patton DU, et al. Considerations for development of child abuse and neglect phenotype with implications for reduction of racial bias: a qualitative study. Journal of the American Medical Informatics Association : JAMIA. 2022;29(3):512-9. doi: 10.1093/jamia/ocab275.

Martin K, Stanford C. An analysis of documentation language and word choice among forensic mental health nurses. International journal of mental health nursing. 2020;29(6):1241-52. doi: 10.1111/inm.12763.

Park J, Saha S, Chee B, Taylor J, Beach MC. Physician Use of Stigmatizing Language in Patient Medical Records. JAMA network open. 2021;4(7):e2117052. doi: 10.1001/jamanetworkopen.2021.17052.

Sun M, Oliwa T, Peek ME, Tung EL. Negative Patient Descriptors: Documenting Racial Bias In The Electronic Health Record. Health Aff (Millwood). 2022;41(2):203-11. doi: 10.1377/hlthaff.2021.01423.
