# Supplementary material for: Identifying stigmatizing language in clinical documentation: A scoping review of emerging literature
Source: PLoS One. 2024 Jun 28;19(6):e0303653. doi: 10.1371/journal.pone.0303653 (PMC11213326; doi:10.1371/journal.pone.0303653)
Supplement: S1 Table — (DOCX) [file pone.0303653.s003.docx]

**S1 Table. Search strategy**

| **Concepts** | **Related terms for search** |
| --- | --- |
| Stigmatizing Language | Stigmatizing language, bias, stigma, linguistic bias |
| Clinician | Provider, clinician, doctor, physician, nurse |
| Clinical Notes | Clinical notes, electronic health records, EHR, medical records |
| **Database** | **Search terms** |
| PubMed | (stigma*[All Fields] OR bias* [All Fields]) AND (language* OR linguistic*) AND (provider* OR clinician* OR doctor* OR physician* OR nurse*) AND (communicat*[All Fields] OR document*[All Fields] OR "clinical note*"[All Fields] OR "Electronic health record*"[All Fields] OR "EHR"[All Fields] OR "Electronic Medical Record*"[All Fields] OR "Medical Record*"[All Fields] OR "Health Record*"[All Fields]) |
| CINAHL | (stigma* OR bias*) AND (language* OR linguistic*) AND (provider* OR clinician* OR doctor* OR physician* OR nurse*) AND (communicat* OR document* OR "clinical note*" OR "Electronic health record*" OR "EHR" OR "Electronic Medical*” OR “Health Record*” OR “medical record*”) |
| Embase | (stigma* OR bias*) AND (language* OR linguistic*) AND (provider* OR clinician* OR doctor* OR physician* OR nurse*) AND (communicat* OR document* OR "clinical note*" OR "Electronic health record*" OR "EHR" OR "Electronic Medical*” OR “Health Record*” OR “medical record*”) |
